# Early Balloon Kyphoplasty Treatment for Osteoporotic Vertebral Fracture Reduces Adjacent Vertebral Fractures

**DOI:** 10.3390/medicina60071097

**Published:** 2024-07-04

**Authors:** Hiromitsu Takano, Hidetoshi Nojiri, Arihisa Shimura, Juri Teramoto, Yuta Sugawara, Muneaki Ishijima

**Affiliations:** 1 Department of Orthopedic Surgery, Juntendo University, Tokyo 113-8421, Japan; hrtakano@juntendo.ac.jp (H.T.); a-shimura@juntendo.ac.jp (A.S.); j-ooka@juntendo.ac.jp (J.T.); y.sugawara.ke@juntendo.ac.jp (Y.S.); ishijima@juntendo.ac.jp (M.I.); 2 Spine and Spinal Cord Center, Juntendo University Hospital, Tokyo 113-8421, Japan

**Keywords:** osteoporotic vertebral fracture, balloon kyphoplasty, adjacent vertebral fracture, secondary vertebral fracture

## Abstract

*Background and Objectives*: This study retrospectively examined whether the incidence rates of adjacent vertebral fractures (AVFs) can be reduced through balloon kyphoplasty (BKP) for osteoporotic vertebral fractures (OVFs) in the early stages, when there is little vertebral height variation. *Materials and Methods*: A total of 95 patients (22 males, 73 females, mean age: 80.7 years) who had undergone BKP were divided into two groups: the Early group (underwent BKP within 2 weeks after injury, *n* = 62), and the Non-early group (underwent BKP > 2 weeks after injury, *n* = 33). The following data were analyzed: patient characteristics; fracture level; the presence of old vertebral fractures, posterior wall injury, and intravertebral cleft; duration of surgery; duration of hospitalization; cement volume; the occurrence of AVF; the timing of AVF occurrence; Numerical Rating Scale (NRS) scores at the preoperative, postoperative, and final follow-up assessments; posterior vertebral kyphosis angle of the affected vertebra on plain lateral X-ray; vertebral wedge ratio; local kyphotic angle; and changes in posterior vertebral kyphosis angle, vertebral wedge ratio, and local kyphotic angle between preoperative and postoperative assessments. The patients were divided based on the occurrence or non-occurrence of AVF after BKP: the Non-AVF group, in which AVF did not occur, and the AVF group, in which AVF occurred. *Results*: The incidence of AVF was 15.8% (15/95 patients), with a notably lower incidence rate in the Early group at 6.5% (4/62 patients) compared to the Non-early group at 33.3% (11/33 patients). NRS scores significantly improved in both groups at the postoperative assessment and final follow-up. The changes in posterior vertebral kyphosis angle and vertebral wedge ratio were significantly lower in the Early group. In the Non-AVF group, the time from injury to surgery was significantly shorter. *Conclusions*: The Early group had a significantly lower incidence of AVF. The time from injury to surgery was a risk factor for AVF occurrence, suggesting that early BKP is recommended.

## 1. Introduction

With the emergence of an ultra-aging society, the frequency of osteoporotic vertebral fractures (OVFs) is increasing. OVF affects life expectancy. The 5-year survival rate after OVF is approximately 60%, compared to 40–50% after femoral neck fractures [1]. Moreover, the relative risk of mortality following a fracture occurrence is reported to be 8.6 times higher for vertebral fractures and 6.7 times higher for femoral neck fractures [2]. Treatment for OVF is primarily conservative but is associated with decreased activities of daily living (ADL) and disuse associated with conservative therapy, residual pain due to vertebral compression, pseudoarthrosis, and delayed neurological impairment. As surgical treatments for OVF, minimally invasive procedures like balloon kyphoplasty (BKP), lateral lumbar interbody fusion (LLIF), and anterior vertebral replacement using X-core2 are highly effective. Since BKP has been covered by insurance in Japan since 2011, its use for OVF has become widespread domestically. BKP is a highly advantageous technique characterized by its minimally invasive nature (short surgical duration, small incision, and minimal blood loss) and outcomes like early pain relief, early mobilization, and short hospital stays, with significant complications being rare. BKP, aimed at early pain relief, early mobilization and return to social activities, prevention of further vertebral compression at the fracture site, and maintenance of spinal alignment, is an actively pursued and effective intervention [3,4]. However, postoperative adjacent vertebral fractures (AVFs) may occur [5,6,7], which can lead to recurrent low back pain, decreased ADL, and disruption of local kyphotic correction, thereby posing issues related to spinal alignment [8]. As many factors contributing to AVF remain unclear, this study retrospectively examined whether performing BKP for an OVF with a minimal vertebral height change in the early stages could reduce the incidence of AVF.

## 2. Materials and Methods

**Figure 1:**
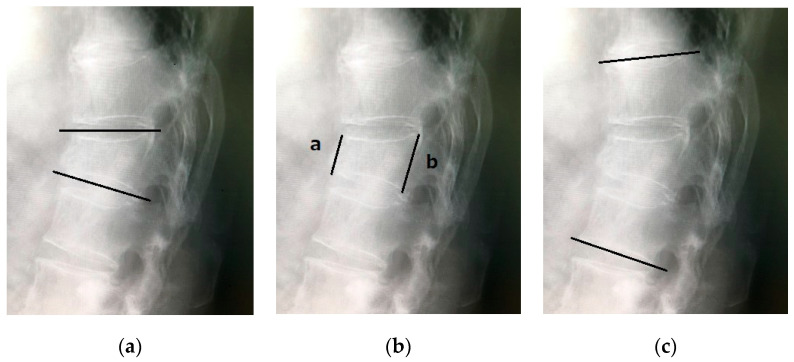
(**a**) Vertebral kyphosis angle; (**b**) vertebral wedge ratio (a/b × 100); and (**c**) local kyphosis angle.

This study included 95 patients (22 males, 73 females) who had undergone BKP at our institution or affiliated hospitals outside the authors’ affiliation between 2021 and 2022 and were followed up for over 1 year postoperatively. The patients were divided into two groups: BKP within 2 weeks after injury (Early group) and BKP > 2 weeks after injury (Non-early group). The date of injury was defined as the date of low back pain or the date of the inferred injury mechanism that caused the OVF, followed by MRI evaluation of a new OVF. Patients with steroid-induced osteoporosis, pathological fractures, traumatic fractures, and diffuse idiopathic skeletal hyperostosis (DISH) were excluded. All patients wore a soft corset for 3 months postoperatively, and osteoporosis treatment, such as parathyroid hormone analogs, anti-sclerostin antibody agents, and anti-RANKL antibody agents, was introduced whenever possible. This study investigated the following parameters: preoperative patient characteristics; fracture level; the presence of old vertebral fractures; the presence of posterior wall injury; the presence of intravertebral cleft; duration of surgery; duration to hospitalization; cement volume; the occurrence of AVF; the timing of AVF occurrence; Numerical Rating Scale (NRS) scores at preoperative, postoperative, and final follow-up assessments; posterior vertebral kyphosis angle of the affected vertebra on plain lateral X-ray; vertebral wedge ratio; local kyphotic angle; and changes in each of these parameters between preoperative and postoperative assessments (Figure 1). Statistical analysis was conducted separately for the Early and Non-early groups using the Mann–Whitney U test, with *p* < 0.05 considered statistically significant. After univariable analysis, those with *p*-values less than 0.1 were selected as covariates. A Variance Inflation Factor (VIF) greater than 10 was excluded to exclude multicollinearity among variables. Multivariable logistic regression analysis was performed to evaluate the risk of AVF occurrence. The research ethics committee at the Faculty of Medicine, Juntendo University, waived the need for ethics approval and the need to obtain consent for the collection, analysis, and publication of the retrospectively obtained and anonymized data for this noninterventional study.

## 3. Results

### 3.1. Patient Characteristics

The mean age was 80.7 years (range, 62–94 years). Among them, 62 patients underwent BKP within 2 weeks after injury (Early group), while 33 patients underwent BKP > 2 weeks after injury (Non-early group). There were 62 cases in the Early group and 33 cases in the Non-early group. The mean age was 81.8 ± 7.3 years in the Early group and 78.5 ± 8.2 years in the Non-early group. The sex ratio (males to females) was 15:47 in the Early group and 7:26 in the Non-early group. There were no cases (0%) of thoracic spine fractures (-T10) in either the Early or Non-early group. Regarding thoracolumbar spine fractures (T11–L2), there were 50 cases (80.6%) in the Early group and 27 cases (81.8%) in the Non-early group. Regarding lumbar spine fractures (L3–L5), there were 12 cases (19.4%) in the Early group and 6 cases (18.2%) in the Non-early group. The presence of old vertebral fractures was observed in 16 cases (25.8%) in the Early group and 14 cases (42.4%) in the Non-early group. Posterior wall injury was present in 25 cases (40.3%) in the Early group and in 14 cases (42.4%) in the Non-early group. Intravertebral cleft was present in 17 cases (27.4%) in the Early group and in 12 cases (36.3%) in the Non-early group. The preoperative NRS score was 9.2 ± 0.7 in the Early group and 9.0 ± 0.7 in the Non-early group, with no significant difference observed. The time from injury to surgery (days) was 8.2 ± 3.0 in the Early group and 46.5 ± 34.1 in the Non-early group, with a significant difference observed in favor of the Early group (*p* < 0.05; Table 1).

### 3.2. Outcomes

**Figure 2:**
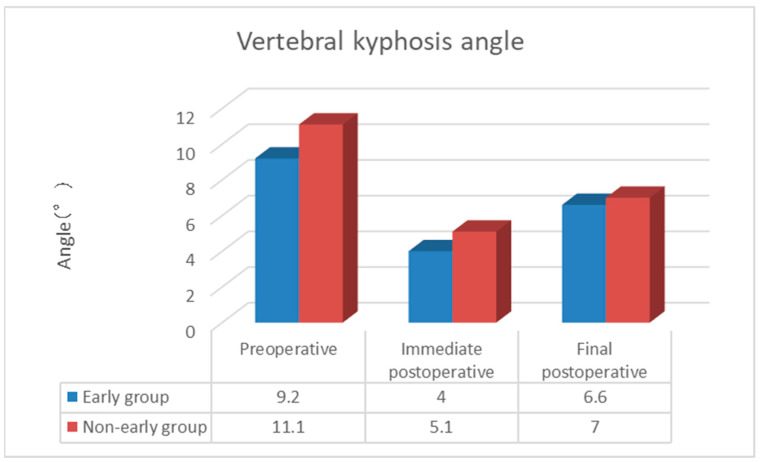
Vertebral kyphosis angle.

**Figure 3:**
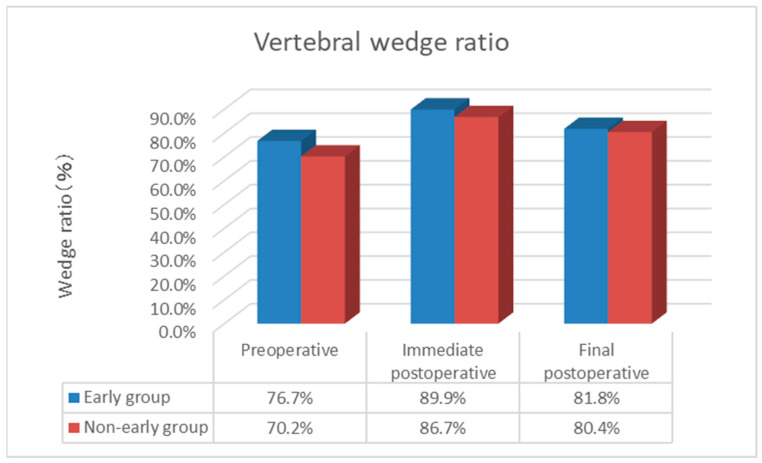
Vertebral wedge ratio.

**Figure 4:**
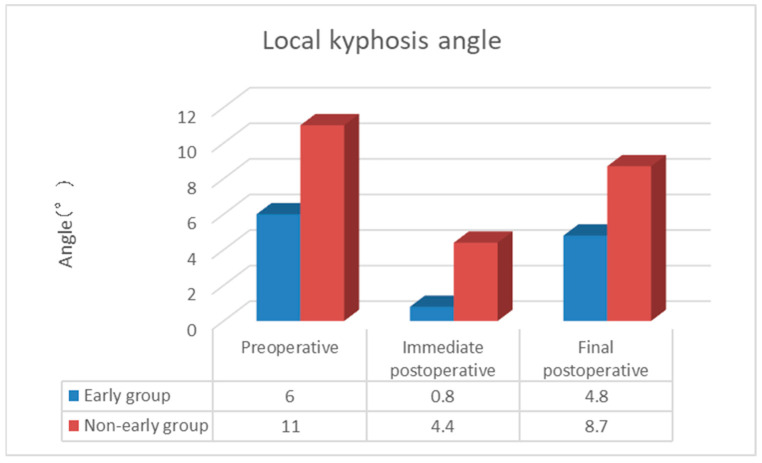
Local kyphosis angle.

The duration of surgery (minutes) was 24.0 ± 2.4 in the Early group and 23.5 ± 2.5 in the Non-early group, with no complications related to general anesthesia or surgical technique, and the amount of bleeding was minimal and immeasurable. The duration of hospitalization (days) was 32.8 ± 15.7 in the Early group and 29.2 ± 17.7 in the Non-early group, while the cement volume (mL) was 7.65 ± 1.37 in the Early group and 7.42 ± 1.73 in the Non-early group, with no significant difference between the two groups. The postoperative NRS score was 0.4 ± 0.7 in the Early group and 0.8 ± 1.3 in the Non-early group, with no significant difference observed between the two groups. The incidence of AVF was 15.8% (15/95 cases), with a significantly lower rate in the Early group at 6.5% (4/62 cases) compared to the Non-early group at 33.3% (11/33 cases) (*p* < 0.05). The time to AVF occurrence (weeks) was 6.5 ± 1.8 in the Early group and 4.1 ± 0.9 in the Non-early group, with AVF occurring significantly earlier in the Non-early group (*p* < 0.05; Table 2). In terms of NRS, in the Early group, scores improved substantially from preoperative (9.2) to postoperative (0.4) assessments and final follow-up (0.4), while in the Non-early group, scores improved similarly from preoperative (9.0) to postoperative (0.8) assessments and final follow-up (0.8). In terms of evaluation on plain lateral X-ray images, the posterior vertebral kyphosis angle in the Early group changed from 9.2° (95% confidence interval (CI) 7.8–10.5) preoperatively to 4.0° (95% CI 3.2–4.8) postoperatively to 6.6° (95% CI 5.5–7.7) at the final follow-up. The change in posterior vertebral kyphosis angle from preoperative to postoperative was 5.2° (95% CI 4.2–6.2) in the Early group. In the Non-early group, the posterior vertebral kyphosis angle changed from 11.1° (95% CI 9.6–12.6) preoperatively to 5.1° (95% CI 3.5–6.8) postoperatively to 7.0° (95% CI 5.1–9.0) at the final follow-up. The change in posterior vertebral kyphosis angle from preoperative to postoperative was 6.0° (95% CI 4.2–6.9) in the Non-early group. The vertebral wedge ratio in the Early group changed from 76.7% preoperatively to 89.9% postoperatively to 81.8% at the final follow-up, with a change of 13.2% from preoperative to postoperative assessments. In the Non-early group, the vertebral wedge ratio changed from 70.2% preoperatively to 86.7% postoperatively to 80.4% at the final follow-up. The changes in both posterior vertebral kyphosis angle and vertebral wedge ratio were significantly lower in the Early group (*p* < 0.05; Figure 2 and Figure 3). The local kyphotic angle changed from 6.0° (95% CI 2.4–9.6) preoperatively to 0.8° (95% CI −2.4–3.9) postoperatively to 4.8° (95% CI 1.4–8.2) at the final follow-up in the Early group. In the Non-early group, the local kyphotic angle changed from 11.0° (95% CI 6.2–15.8) preoperatively to 4.4° (95% CI 0.4–8.4) postoperatively to 8.7° (95% CI 3.9–13.5) at the final follow-up, with no significant difference between the two groups (Figure 4). AVF occurred in 15.8% (15/95 cases), and in the univariate analysis, where patients were divided into the Non-AVF group (80 cases) and the AVF group (15 cases), no significant differences were found in age, presence of old vertebral fracture, cement volume, change in posterior vertebral kyphosis angle, change in vertebral wedge ratio, or change in postoperative local kyphotic angle. However, significant differences were observed in time from injury to surgery, preoperative posterior vertebral kyphosis angle, postoperative posterior vertebral kyphosis angle, final follow-up posterior vertebral kyphosis angle, preoperative vertebral wedge ratio, postoperative vertebral wedge ratio, final follow-up vertebral wedge ratio, preoperative local kyphotic angle, final follow-up local kyphotic angle, and change in local kyphotic angle (*p* < 0.05; Table 3). Table 4 shows the adjusted odds ratios for time from injury to surgery, preoperative posterior vertebral kyphosis angle, and change in local kyphotic angle with respect to AVF. The adjusted odds ratios for Early BKP or Non-early BKP, change in vertebral wedge ratio, preoperative vertebral wedge ratio, and preoperative local kyphosis angle were 7.663 (95% confidence interval (CI) 1.802–32.582; *p* = 0.006), 1.058 (95% CI 0.977–1.145; *p* = 0.164), 1.167 (95% CI 1.036–1.315; *p* = 0.011), and 0.777 (95% CI 0.571–1.057; *p* = 0.108), respectively. This suggests that Early BKP or Non-early BKP is associated with a higher risk of AVF occurrence.

## 4. Discussion

Previously, the treatment principle for OVF leaned towards conservative treatment, with orthosis therapy being a common choice. However, regardless of whether rigid or soft corsets were used, there was no significant difference in preventing vertebral deformity, improving QOL, or providing pain relief, with approximately 36% of OVF cases progressing to progressive vertebral compression and around 14% developing pseudoarthrosis [9,10]. Moreover, 21.3% of cases experienced a decline in ADL 6 months after injury [11]. Hence, there are cases where early surgery is desirable. BKP enables early mobilization due to early pain relief and reduces mortality rates [12]. Early BKP is recommended for cases of OVF with poor pain improvement because it not only improves pain but also helps maintain ADL and prevents medical complications [13,14,15]. Nowadays, there is a tendency to perform early BKP, especially when magnetic resonance imaging (MRI) shows prognostic factors like extensive T1 hypointensity, T2 hypointensity, or localized T2 hyperintensity [16]. However, even in cases of OVF considered to have a good prognosis on MRI, residual lower back pain was observed in 29.1% of cases at 6 months, indicating a poor prognosis [17]. The study was not randomized, and all new OVF patients with pain were treated with BKP. The incidence of AVF was reported to be around 14–35.7% [5,6,18,19]; in this study, the AVF incidence was 15.8%, which is consistent with previous reports. However, in the Early group, the AVF incidence was significantly lower at 6.5% compared to the Non-early group. The 1-year repeat fracture rate in the natural course after an OVF is 19.2% [19,20], and the AVF rate is not necessarily higher than the postoperative AVF rate after BKP, suggesting that early BKP may prevent secondary vertebral fractures. In addition, it has been reported that postoperative AVF is a risk factor for prolonged pain and reoperation [21,22]. Postoperative AVF after BKP often occurs within 2 months [7]. When comparing the incidence of AVF after BKP, it is not necessarily higher. On the contrary, some have argued that performing early BKP may prevent subsequent vertebral fractures. Early BKP not only enables an early return to society but also helps maintain spinal alignment and reduces the occurrence of secondary fractures like AVF [4]. The AVF risk consists of five preoperative factors: (1) intravertebral instability (≥5 mm), (2) focal kyphosis (≥10°), (3) duration of symptoms (≥30 days), (4) intravertebral cleft, and (5) previous history of vertebral fracture [23]. The risk factors for AVF include excessive correction of kyphosis and residual kyphotic deformity [24]. In OVF, if treated early, there is minimal change in vertebral height and maintenance of vertebral height, whereas delayed treatment often leads to significant changes in vertebral height and worsening alignment. Our treatment strategy for OVF involves performing BKP in all cases of new OVF, regardless of the prognostic factors on MRI. Early BKP for OVF is controversial because the treatment principle for OVF is conservative treatment. Even in cases that would have been cured by BKP in the early stage, patients may choose conservative treatment, resulting in their vertebrae being crushed, and requiring major surgery such as an anterior vertebral body replacement or posterior osteotomy. In addition, most patients are elderly, and the role of early BKP to immediately relieve pain and improve ADL is very significant. In this study, the changes in vertebral kyphotic angle and vertebral wedge ratio were lower in the Early group, suggesting that early BKP reduces changes in vertebral height before and after surgery, decreases stress on adjacent vertebrae, and consequently reduces the incidence of AVF. On the other hand, it has been reported that BKP of a single vertebral body corrects local kyphosis but does not have the ability to correct global alignment [25]. Local kyphosis correction also fails when AVF occurs [8]. Anabolic agents could improve pain and strongly reduce the risk of upcoming subsequent fractures [26,27]. In this study, anabolic agents were also introduced after BKP to the extent possible. Although percutaneous vertebroplasty (PVP) is considered a relative contraindication to treatment of vertebral plana, good results have been reported with reduction in pain by percutaneous cement augmentation [28,29]. In this study, BKP was also performed on vertebral plana that had remaining space that could be filled with cement, with good results. However, for vertebral plana that are severely crushed and difficult to cement, anterior reconstruction such as an anterior vertebral body replacement or posterior osteotomy may be necessary, and careful consideration is required. The reason why the average length of hospitalization was as long as one month was because all cases in this study were at a community-based regional hospital, and many elderly patients tended not to wish to be discharged early but to stay for a long period of time, including rehabilitation. A limitation of this study is the lack of long-term follow-up, which prevents the evaluation of the frequency of AVF and clinical outcomes after the study period. The follow-up period may also vary from case to case. However, AVF after BKP often occurs within 2 months postoperatively [7], and therefore, the results of this study are considered sufficiently informative. Furthermore, all cases in this study were BKP cases and did not include cases treated conservatively. Although BMD assessment is extremely important when BKP is performed [30], in this study, BMD assessment was performed using radial DXA and was not performed in all cases, so it was omitted from the study items. Additionally, strong preoperative pain might have hindered the evaluation of overall spinal alignment. Moving forward, extending the study period and increasing the number of cases for further examination are warranted.

## 5. Conclusions

We divided patients into an Early group, who had undergone BKP within 2 weeks after injury, and a Non-early group, who had undergone BKP > 2 weeks after injury, to retrospectively investigate whether performing BKP early with minimal vertebral height change reduces the incidence of AVF. The AVF incidence was lower in the Early group compared to the Non-early group, and further analysis of the AVF incidence revealed that the time from injury to surgery was a risk factor for AVF development. We recommend performing BKP early with minimal vertebral height change for OVF. A multidisciplinary and tailored approach remains the best manner to obtain long-term benefits for osteoporotic patients in terms of primary and secondary prevention of fragility fractures [31,32].

## Figures and Tables

**Table 1:** Preoperative demographic characteristics of the patients in each group.

	Early Group (*n* = 62)	Non-Early Group (*n* = 33)	*p*-Value
Age (years)	81.8 ± 7.3	78.5 ± 8.2	N.S.
Sex (male/female)	M15/F47	M7/F26	N.S.
Time from injury to surgery (days)	8.2 ± 3.0	43.2 ± 34.1	*p* < 0.05
Fracture level			
Thoracic spine (-T10)	0 (0%)	0(0%)	N.S.
Thoracolumbar spine (T11–L2)	50 (80.6%)	27 (81.8%)	N.S.
Lumbar spine (L3–L5)	12 (19.4%)	6 (18.2%)	N.S.
Old vertebral fracture			
0	46 (74.2%)	19 (57.6%)	N.S.
≧1	16 (25.8%)	14 (42.4%)	N.S.
Posterior wall injury (%)	25 (40.3%)	14 (42.4%)	N.S.
Intravertebral cleft	17 (27.4%)	12 (36.3%)	N.S.
Preoperative NRS	9.2 ± 0.7	9.0 ± 0.7	N.S.

N.S.: not significant.

**Table 2:** Postoperative findings of the patients in each group.

	Early Group (*n* = 62)	Non-Early Group (*n* = 33)	*p*-Value
Duration of surgery (min)	24.0 ± 2.4	23.5 ± 2.5	N.S.
Duration to hospitalization (days)	32.8 ± 15.7	29.2 ± 17.7	N.S.
Cement volume (mL)	7.65 ± 1.37	7.42 ± 1.73	N.S.
AVF	4 (6.5%)	11 (33.3%)	*p* < 0.05
Duration to AVF (weeks)	6.5 ± 1.8	4.1 ± 0.9	*p* < 0.05
Postoperative NRS	0.4 ± 0.7	0.8 ± 1.3	N.S.

N.S.: not significant.

**Table 3:** Univariable analysis between groups with and without AVF.

	Non-AVF Group (*n* = 80)	AVF Group (*n* = 15)	*p*-Value
Age (years)	81.2 ± 5.3	77.7 ± 9.3	N.S.
Time from injury to surgery (days)	15.8 ± 20.1	44.5 ± 38.6	0.001
Old vertebral fracture	0.44 ± 0.83	0.8 ± 0.9	N.S.
Cement volume (mL)	7.63 ± 1.43	7.27 ± 1.84	N.S.
Preoperative vertebral kyphosis angle	−9.2 ± 5.3	−13.3 ± 5.8	0.009
Immediate postoperative vertebral kyphosis angle	−4 ± 3.5	−6.3 ± 4.7	0.033
Final postoperative vertebral kyphosis angle	−6.2 ± 4.5	−9.7 ± 4.5	0.008
Change in vertebral kyphosis angle	5.1 ± 3.2	6.3 ± 3.1	N.S.
Preoperative vertebral wedge ratio (%)	77.0 ± 14.1	61.1 ± 17.4	0.001
Immediate postoperative vertebral wedge ratio (%)	90.1 ± 8.9	81.5 ± 13.6	0.003
Final postoperative vertebral wedge ratio (%)	83.3 ± 12.2	72.7 ± 12.6	0.001
Change in vertebral wedge ratio (%)	12.9 ± 10.1	20.4 ± 11.4	N.S.
Preoperative local kyphosis angle	−6.4 ± 13.3	−14.9 ± 15.4	0.032
Immediate postoperative local kyphosis angle	−1.2 ± 11.6	−6.4 ± 12.7	N.S.
Final postoperative local kyphosis angle	−4.8 ± 12.8	−12.9 ± 13.8	0.020
Change in local kyphosis angle	4.9 ± 4.3	8.4 ± 5.6	0.025

N.S.: not significant.

**Table 4:** Multivariable logistic regression for AVF.

Risk Factor	Odds Ratio (95% Confidence Interval)	*p*-Value
Early BKP or Non-early BKP	7.663 (1.802 to 32.582)	0.006
Change in vertebral wedge ratio	1.058 (0.977 to 1.145)	0.164
Preoperative vertebral wedge ratio	1.167 (1.036 to 1.315)	0.011
Preoperative local kyphosis angle	0.777 (0.571 to 1.057)	0.108

## Data Availability

Due to privacy and ethical constraints, individual data cannot be disclosed.

## References

[1] Tsuboi M., Hasegawa Y., Suzuki S., Wingstrand H., Thorngren K.G. (2007). Mortality and mobility after hip fracture in Japan: A ten-year follow-up. J. BoneJoint Surg. Br..

[2] Cauley J.A., Thompson D.E., Ensrud K.C., Scott J.C., Black D. (2000). Risk of mortality following clinical fractures. Osteoporos. Int..

[3] Wardlaw D., Cummings S.R., Meirhaeghe J.V., Bastian L., Tillman J.B., Ranstam J., Eastell R., Shabe P., Talmadge K., Boonen S. (2009). Efficacy and safety of balloon kyphoplasty compared with nonsurgical care for vertebral compression fracture (FREE): A randomised controlled trial. Lancet.

[4] Minamide A., Maeda T., Yamada H., Murakami K., Okada M., Enyo Y., Nakagawa Y., Iwasaki H., Tsutsui S., Takami M. (2018). Early varsus delayed kyphoplasty for thoracolumbar osteoprotic verterbral fractures: The effect of timing on clinical and radiographic outcomes and subsequent compression fractures. Clin. Neurol. Neurosurg..

[5] Trout A.T., Kallmes D.F., Lane J.I., Layton K.F., Marx W.F. (2006). Subsequent vertebral fractures after vertebroplasty association with intraosseous clefts. Am. J. Neuroradial..

[6] Flankel B.M., Monroe T., Wang C. (2007). Percutaneous vertebral augmentation: An elevation in adjacent-level fracture risk in kyphoplasty as compared with vertebroplasty. Spine J..

[7] Fribourg D., Tang C., Sra P., Delamarter R., Bae H. (2004). Incidence of subsequent vertebral fracture after kyphoplasty. Spine.

[8] Iida k., Harimaya K., Tarukado K., Tono O., Matsumoto Y., Nakashima Y. (2019). Kyhosis progression after balloon kyphoplasty compared with conservative treatment. Asian Spine J..

[9] Grafe I.A., Fonseca D.K., Hillmeier J., Meeder P.J., Libicher M., Nöldge G., Bardenheuer H., Pyerin W., Basler L., Weiss C. (2005). Reduction of pain and fracture incidence after kyphoplasty: 1-year outcomes of a prospective controlled trial of patients with primary osteoporosis. Osteoporos. Int..

[10] Kato T., Inose H., Ichimura S., Tokuhashi Y., Nakamura H., Hoshino M., Togawa D., Hirano T., Haro H., Ohba T. (2019). Comparison of Rigid and Soft-Brace Treatments for Acute Osteoporotic Vertebral Compression Fracture: A Prospective, Randomized, Multicenter Study. J. Clin. Med..

[11] Matsumoto T., Hoshino M., Tsujio T., Terai H., Namikawa T., Matsumura A., Kato M., Toyoda H., Suzuki A., Takayama K. (2012). Prognostic factors for reduction of activities of daily living following osteoporotic vertebral fractures. Spine.

[12] Phillis F.M., Ho E., Campbell-Hupp M., McNally T., Wetzel F.T., Gupta P. (2003). Early radiographic and clinica results of balloon kyphoplasty for the treatment of osteoporotic vertebral compression fractures. Spine.

[13] Takahashi S., Hoshino M., Terai H., Toyoda H., Suzuki A., Tamai K., Watanabe K., Tsujio T., Yasuda H., Kono H. (2018). Differences in short-term clinical and radiological outcomes depending on timing of balloon kyphoplasty for painful osteoporotic vertebral fracture. J. Orthop. Sci..

[14] Sakai T. (2018). Is Early Stage Balloon Kyphoplasty for Osteoporotic Vertebral Fracture Appropriate?. J. Spine Res..

[15] Shawn J.S., Mark H.Y., Jun-Hao T., Hwee Weng D.H. (2023). Early cement augmentation may be a good treatment option for pain relief for osteoporotic compression fractures: A systematic review and meta-analysis. Eur. Spine J..

[16] Tsujio T., Nakamura H., Terai H., Hoshino M., Namikawa T., Matsumura A., Kato M., Suzuki A., Takayama K., Fukushima W. (2011). Characteristic radiographic or magnetic resonance images of fresh osteoporotic vertebral fractures predicting potential risk for nonunion: A prospective multicenter study. Spine.

[17] Iwamae M., Terai H., Tamai K., Hoshino M., Takahashi S., Umano M., Kobayashi Y., Katsuda H., Kaneda K., Shimada N. (2023). Risk of residual low back pain in conservative treatment of osteoporotic vertebral fractures without poor prognostic factors on magnetic resonance imaging. J. Spine Res..

[18] Zhang T., Wang Y., Zhang P., Xue F., Zhang D., Jiang B. (2022). What are the risk factors for adjacent vertebral fracture after vertebral augmentation? A meta-analysis of published studies. Glob. Spine J..

[19] Lindsay R., Silverman S.L., Cooper C., Hanley D.A., Barton I., Broy S.B., Licata A., Benhamou L., Geusens P., Flowers K. (2001). Risk of new vertebral fracture in the year following a fracture. JAMA.

[20] Kadowaki E., Tamaki J., Iki M., Sato Y., Chiba Y., Kajita E., Kagamimori S., Kagawa Y., Yoneshima H. (2010). Prevalent vertebral deformity independently increases incident vertebral fracture risk in middle-aged and elderly Japanese women: The Japanese Population-based Osteoporosis (JPOS) Cohort Study. Osteoporos. Int..

[21] Yang S.C., Chen W.J., Yu S.W., Tu Y.K., Kao Y.H., Chung K.C. (2008). Revision strategies for complications and failure of vertebroplasties. Eur. Spine J..

[22] Takahara K., Kamimura M., Moriya H., Ashizawa R., Koike T., Hidai Y., Ikegami S., Nakamura Y., Kato H. (2016). Risk factors of adjacent vertebral collapse after percutaneous vertebroplasty for osteoporotic vertebral fracture in postmenopausal women. BMC Muscoskel. Disord..

[23] Hijikata Y., Kamitani T., Nakahara M., Kumamoto S., Sakai T., Itaya T., Yamazaki H., Ogawa Y., Kusumegi A., Inoue T. (2022). Development and internal validation of a clinical prediction model for acute adjacent vertebral fracture after vertebral augmentation. Bone Joint J..

[24] Morozumi M., Matsubara Y., Muramoto A., Morita Y., Ando K., Kobayashi K., Machino M., Ota K., Tanaka S., Kanbara S. (2020). A study of risk factors for early-onset adjacent vertebral fractures after kyphoplasty. Glob. Spine J..

[25] Paradhan B.B., Bae H.W., Kropf M.A., Patel V.V., Delamarter R.B. (2006). Kyphoplasty reduction of osteoporotic vertebral compression fractures correction of local kyphosis versus overall sagittal alignment. Spine.

[26] Langdahl B.L., Rajzbaum G., Jakob F., Karras D., Ljunggren O., Lems W.F., Fahrleitner-Pammer A., Walsh J.B., Barker C., Kutahov A. (2009). Reduction in fracture rate and back pain and increased quality of life in postmenopausal women treated with teriparatide: 18-month data from the European Forsteo Observational Study (EFOS). Calcif. Tissue Int..

[27] Chen Z., Lin W., Zhao S., Mo X., Yuan W., Cheung W.H., Fu D., Chen B. (2021). Effect of Teriparatide on pain relief, and quality of life in postmenopausal females with osteoporotic vertebral compression fractures, a retrospective cohort study. Ann. Palliat. Med..

[28] Joyce D.M., Granville M., Berti A., Jacobson R.E. (2022). Vertebral Augmentation Compared to Conservative Treatment of Vertebra Plana and High-Degree Osteoporotic Vertebral Fractures: A Review of 110 Fractures in 100 Patients. Cureus.

[29] Pedicelli A., Lozupone E., Gatto A., Gulino P., D’Argento F., Capozzi A., Colosimo C. (2013). Vertebra plana: Reappraisal of a contraindication to percutaneous vertebroplasty. Eur. J. Radiol..

[30] Lamy O., Uebelhart B., Aubry-Rozier B. (2014). Risks and benefits of percutaneous vertebroplasty or kyphoplasty in the management of osteoporotic vertebral fractures. Osteoporos. Int..

[31] Capozzi A., Scambia G., Pedicelli A., Evangelista M., Sorge R., Lello S. (2016). Clinical management of osteoporotic vertebral fracture treated with percutaneous vertebroplasty. Clin. Cases Miner. Bone Metab..

[32] Taha K.A., Lauper N., Bauer D.F., Tsoupras A., Tessitore E., Biver E., Dominguez D.E. (2024). Multidisciplinary and Coordinated Management of Osteoporotic Vertebral Compression Fractures: Current State of the Art. J. Clin. Med..

